# Author Correction: Ascorbic acid as an adjunctive therapy in critically ill patients with COVID-19: a propensity score matched study

**DOI:** 10.1038/s41598-021-99146-7

**Published:** 2021-09-24

**Authors:** Khalid Al Sulaiman, Ohoud Aljuhani, Khalid Bin Saleh, Hisham A. Badreldin, Abdullah Al Harthi, Mohammed Alenazi, Aisha Alharbi, Rahmah Algarni, Shmeylan Al Harbi, Abdullah M. Alhammad, Ramesh Vishwakarma, Sarah Aldekhyl

**Affiliations:** 1 Pharmaceutical Care Department, King Abdulaziz Medical City, Riyadh, Saudi Arabia; 2 Department of Pharmacy Practice, Faculty of Pharmacy, King Abdulaziz University, Jeddah, Saudi Arabia; 3 Pharmaceutical Care Department, King Abdulaziz University Hospital, Jeddah, Saudi Arabia; 4 College of Medicine, King Saud Bin Abdulaziz University for Health Sciences, King Abdullah International Medical Research Center, Riyadh, Saudi Arabia; 5 Intensive Care Department, King Abdulaziz Medical City, Riyadh, Saudi Arabia; 6 College of Pharmacy, King Saud Bin Abdulaziz University for Health Sciences, Riyadh, Saudi Arabia; 7 Biostatistics and Bioinformatics Department, King Abdullah International Medical Research Center, Riyadh, Saudi Arabia; 8 Department of Clinical Pharmacy, College of Pharmacy, King Saud University, Riyadh, Saudi Arabia; 9 King Abdulaziz Medical City (KAMC)-Ministry of National Guard Health Affairs (MNGHA), King Abdullah International Medical Research Center/King Saud Bin Abdulaziz University for Health Sciences, PO Box 22490, Riyadh, 11426 Saudi Arabia

Correction to: *Scientific Reports* 10.1038/s41598-021-96703-y, published online 03 September 2021

The original version of this Article contained an error in Table 2, where the “Ascorbic acid group, n of outcomes/total no-of patients” for “Ascorbic Acid” was incorrect for the Outcome “Thrombosis during ICU, Analysis on all eligible patients”.

“9/5.8 (2.3)”.

now reads:

“9/154 (5.8)”.

The original Article has been corrected.

